# Molecular response of *Deinococcus radiodurans* to simulated microgravity explored by proteometabolomic approach

**DOI:** 10.1038/s41598-019-54742-6

**Published:** 2019-12-05

**Authors:** Emanuel Ott, Felix M. Fuchs, Ralf Moeller, Ruth Hemmersbach, Yuko Kawaguchi, Akihiko Yamagishi, Wolfram Weckwerth, Tetyana Milojevic

**Affiliations:** 1 Extremophiles/Space Biochemistry Group, Department of Biophysical Chemistry, University of Vienna, Vienna, Austria; 2 Space Microbiology Research Group, Radiation Biology Department, Institute of Aerospace Medicine, German Aerospace Centre, Cologne, Germany; 3 Division of Gravitational Biology, Institute of Aerospace Medicine, German Aerospace Centre, Cologne, Germany; 4 Planetary Exploration Research Centre (PERC), Chiba Institute of Technology (CIT), Chiba, Japan; 5 Department of Life Science and Technology, Tokyo Institute of Technology, Nagatsuta, Yokohama, Japan; 6 Department of Ecogenomics and Systems Biology, University of Vienna, Vienna, Austria; 7 Vienna Metabolomics Centre (VIME), University of Vienna, Vienna, Austria

**Keywords:** Bacterial systems biology, Signal processing

## Abstract

Regarding future space exploration missions and long-term exposure experiments, a detailed investigation of all factors present in the outer space environment and their effects on organisms of all life kingdoms is advantageous. Influenced by the multiple factors of outer space, the extremophilic bacterium *Deinococcus radiodurans* has been long-termly exposed outside the International Space Station in frames of the Tanpopo orbital mission. The study presented here aims to elucidate molecular key components in *D. radiodurans*, which are responsible for recognition and adaptation to simulated microgravity. *D. radiodurans* cultures were grown for two days on plates in a fast-rotating 2-D clinostat to minimize sedimentation, thus simulating reduced gravity conditions. Subsequently, metabolites and proteins were extracted and measured with mass spectrometry-based techniques. Our results emphasize the importance of certain signal transducer proteins, which showed higher abundances in cells grown under reduced gravity. These proteins activate a cellular signal cascade, which leads to differences in gene expressions. Proteins involved in stress response, repair mechanisms and proteins connected to the extracellular milieu and the cell envelope showed an increased abundance under simulated microgravity. Focusing on the expression of these proteins might present a strategy of cells to adapt to microgravity conditions.

## Introduction

Exploration of hostile environments by humans is a dangerous, yet essential and rewarding task with numerous factors to consider. It is of utmost importance to evaluate all environmental factors independently to elaborate their impact on all kind of organisms. Apart from ionizing and solar UV radiation, vacuum, and extreme temperature fluctuations, microgravity is another omnipresent environmental parameter to be considered. As gravity force was always present since the dawn of life, it has influenced the development of all organisms^1^. Numerous studies suggest that microgravity influence proteinaceous cellular components, depending on different cell types^2,3^. In humans, microgravity exposure causes a redistribution of blood toward the head, altered responses of baroreceptor, nervous and endocrine systems, which lead to space motion sickness^4^. In addition, the blood supply to the eye is altered depending on the duration of the flight, which impacts vascularization^5^. Apart from direct effects to animals, microgravity can alter host-symbiont/parasite interactions. A spaceflight experiment was performed with the squid *Euprymna scolopes* and its beneficial symbiont *Vibrio fischeri*. Transcriptomics analyses revealed that under spaceflight conditions, genes associated with oxidative stress response were enriched if the symbiont was absent^6^. Indeed, microgravity can alter cellular interactions between eukaryotes hosts and their associated microbes^7^. Incubation of *E. scolopes* and *V. fischeri* in a high aspect ratio rotating wall vessel resulted in suppression of the host´s innate immune response and acceleration of bacteria-induced apoptosis^8^.

Evolution forced complex organisms to develop systems for fluid regulation, gravity sensing, spatial orientation and locomotion. The STS (Space Transportation System)-95 Space Experiment showed that microgravity does not affect seed germination of pea and maize. However, the orientation of stem elongation, growth and development are strongly affected^9^. On the molecular level, there are indications, that gravity-dependent signal pathways might be controlled by mechano- (gravi-) sensitive ion channels and cascades of ubiquitous second messengers^10^. According to multiple studies, signal amplification, gravity sensing and graviorientation are closely connected to cytoskeletal elements^11–13^ in plants. Single cells may be able to sense changes in gravity and convert them into biochemical signals^14^. These signals may lead to changes in the protein production capability and thus cause increased virulence^15^ or different biofilm production^16^. Investigating infectious organisms under simulated microgravity has revealed an increased production of the heat-labile enterotoxin in *Escherichia coli* and tumour necrosis factor-alpha in the infected murine macrophages^17^. In a similar fashion, simulated microgravity supports the invasive potential of *Salmonella enterica* and enhances the production of tumour necrosis-factor alpha in infected epithelial cells^17^. *Staphylococcus epidermidis* showed an increased mutation of genes connected to resistance to the antibiotic rifampicin 122 h of growth on the ISS^18^. A spaceflight study performed on *E. coli* suggests a connection between increased antibiotic resistance and induction of 50 stress-response genes^19^. Spaceflight induced changes in the proteome of *Pseudomonas aeruginosa* were analysed in a study conducted by Crabbé *et al*.^20^. 28 proteins were identified as differentially expressed with Hfq as a global transcriptional regulator. As Hfq was also differentially expressed in spaceflight-grown *S. enterica*, it represents a spaceflight induced regulator acting across bacterial species^21^. The ∆hfq mutant of *V. fischeri* confirmed that Hfq impacts regulatory processes under low-shear-modelled microgravity (LSMMG) differently than under normal gravity conditions^22^.

Furthermore, in *S. enterica* many ribosomal proteins showed decreased abundance after the spaceflight^21^. In another study, *Rhodospirillum rubrum* showed differentially expressed ribosomal and stress response proteins^23^. Onboard the ISS (International Space Station), *E. coli* cultures showed a 13-fold increase in final cell counts compared to ground control cells^24^. Moreover, *E.coli* was able to grow in presence of normally inhibitory levels of antibiotics such as gentamicin sulphate^24^. The cultures onboard the ISS showed an increase in cell envelope thickness, outer membrane vesicles and tended to form clusters^24^. This aggregation of cells might be associated with effects, observed in biofilm forming bacteria after exposure to microgravity. *Micrococcus luteus*, grown on the ISS, showed an increased production of exopolymeric substances compared to the 1 g ground control strain^25^.

The Gram-positive bacterium *Deinococcus radiodurans* possess some remarkable properties which makes it an ideal candidate for various space-related studies. It is extremely resistant to ionizing radiation^26^, UV radiation^27^ and desiccation^28^. *D. radiodurans* was used in the latest Low Earth orbit exposure mission outside the ISS: the Japanese Tanpopo mission^29^. Many studies were performed to elucidate the mechanisms behind its extraordinary survival regarding ionizing radiation and other reactive oxygen species (ROS) producing environmental factors^30–32^. However, little is known about the molecular response of *D. radiodurans* to microgravity. It was shown that the recovery of *D. radiodurans* after radiation damage is enhanced when subjected to microgravity^33^. Nevertheless, a high-resolution molecular approach, which indicates key components that are responsible for gravity sensing and signal transmission is missing for *D. radiodurans*.

**Figure 1:**
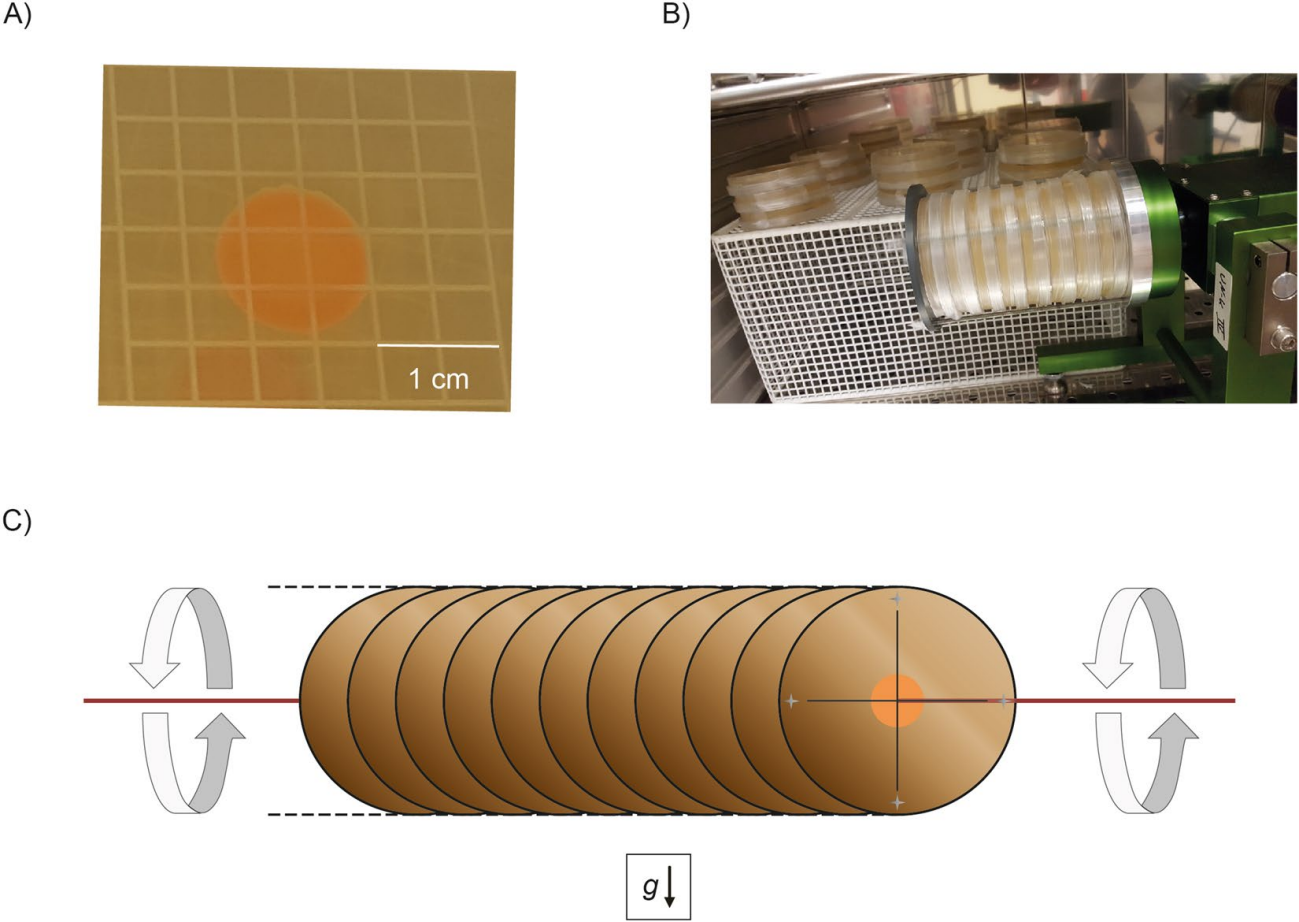
Principle of a 2-D Clinostat: TGB-agar plates are inoculated with 10 µl of an overnight PBS-washed *D. radiodurans* culture using a grid petri dish (**A**). After air-drying for 2 min, plates are sealed with Parafilm, mounted on a 2-D clinostat and secured by a lid and four butterfly screws. The clinostat is aligned so that the axis of rotation is parallel to the ground (**B**). Incubation takes place at 37 °C at 60 rpm, rotating counterclockwise. The scheme in (**C**) represents the principle of a 2D-clinostat: During the entire duration of the experiment, growing *D. radiodurans* cells are exactly placed on the axis of rotation, attached to the centre of a TGB-agar petri dish. Residual acceleration decreased to ~≤0,016 g, when assuming a final colony diameter of 0.8 cm.

In this study, the effects of simulated microgravity on *D. radiodurans* were investigated by growing single cells to colonies during the incubation on a fast-rotating 2-D clinostat (Fig. 1). The 2-D clinostat was used in several microgravity simulation experiments, including *Arabidopsis*^34^ seedlings and *V. natriegens*^35^. Since space experiments require an extraordinary effort due to the planning, cost and experiment design, various ground-based approaches have been developed and are applied over the last centuries^36^. In order to quite simply achieve microgravity (free fall) on Earth only a few methods exist, such as sounding rockets, drop-tower to parabolic flights^37^. Unfortunately, these platforms only grant a little time frame of microgravity in the range of seconds to minutes, in which experiments can be conducted. Thus, facilities have been developed aiming to simulate microgravity for longer periods of time to grow cells for several generations. To simulate a continuous free fall, the rotating wall vessel uses a chamber, completely filled with cells in culture medium. It is rotating around an axle and thus subjecting the cells to a continuous free fall^38^. A random positioning machine consists of two frames (inner and outer frame), rotating independently from each other in random directions. As a consequence, the gravity vector is averaged to zero over time for samples that are located directly in the middle of the machine^39^. The principle of a 2-D clinostat is based on the rotation around a horizontal axis perpendicular to gravity, assuming that cellular gravity-perception does no longer take place^40–42^. In case of agar-based incubation experiments to grow colonies or biofilms, the quality of simulation is strongly limited to the diameter the colony can form. As the diameter increases, residual acceleration increases. Thus, small diameters (*r* ≤ 0.5 cm), and localization of the colony exactly in the centre of rotation within the clinostat were considered. None of these methods achieve gravitational unloading and fluid convection and shear stress are not completely erased, however sedimentation is avoided through the omnilateral gravistimulation^15^. Due to these limitations, results from cells incubated in real microgravity might be similar, but not identical to cells incubated under simulated microgravity^43^.

Following incubation on fast rotating 2-D clinostats, the proteome and metabolome of microgravity-grown cells and 1 g control cells of *D. radiodurans* were analysed. A bottom-up proteomics approach was used as it enables a relative quantitative comparison of a multitude of proteins which might be affected by microgravity. Metabolomics based on a well-established GC-MS approach allowed quantification of polar metabolites. This was performed as a preliminary experiment to real space exposure of *D. radiodurans* in frames of the Tanpopo space mission^29,44,45^. Multiple molecular stress response studies were performed on this well-studied extremophile, resulting in valid annotations for many proteins. Due to its high resistance to various environmental conditions, it is likely that *D. radiodurans* or microorganisms with similar properties are targets for spaceflight missions. Consequently, a proper understanding of how *D. radiodurans* adapt and respond to microgravity as space-environmental conditions is desirable. These results can contribute to understanding how cells react to reduced gravity without other, more influential environmental factors present in Low Earth orbit.

## Results

### Proteomic response to microgravity

**Figure 2:**
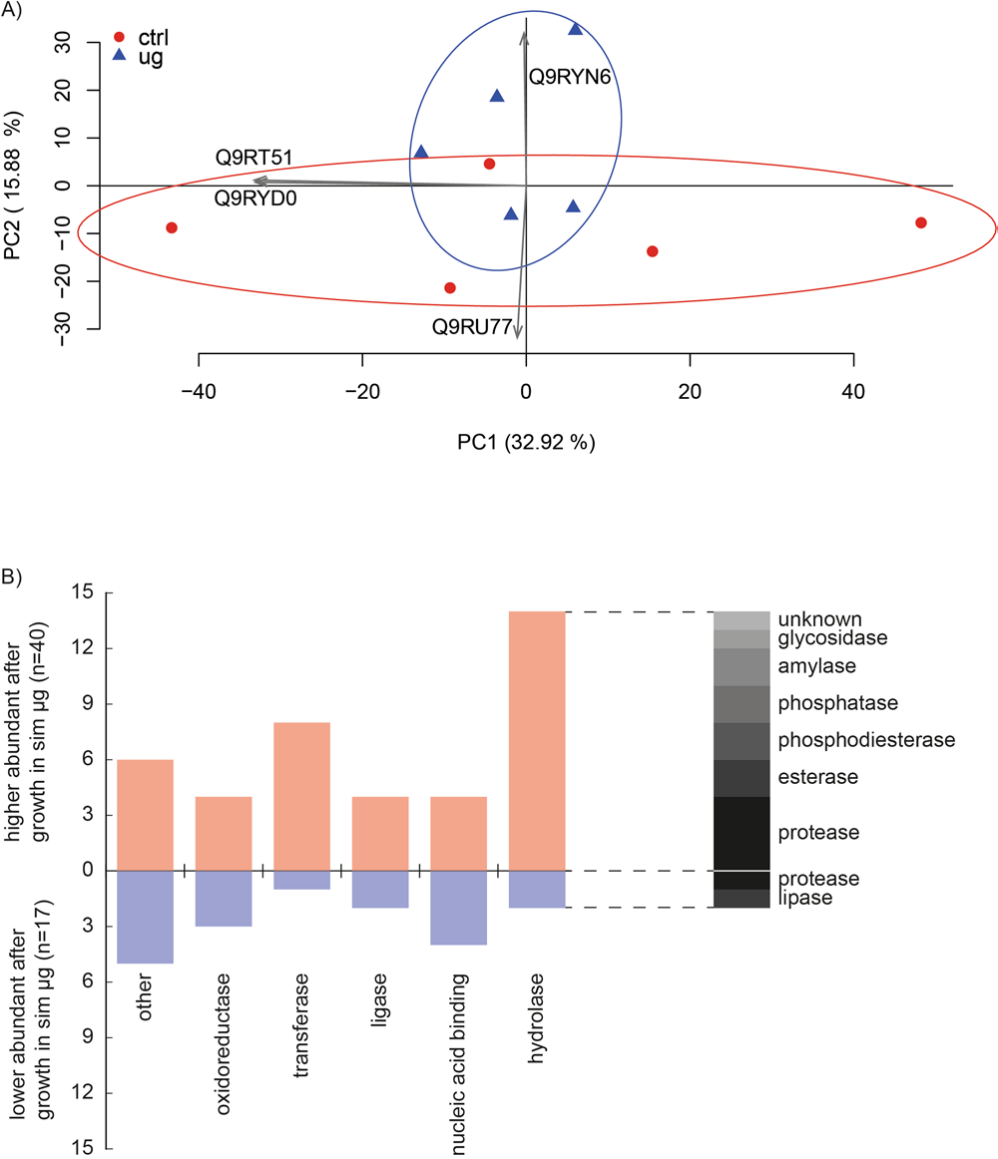
Proteomic response to simulated microgravity. (**A**) PCA of 1618 proteins identified in each of the five replicates in simulated microgravity (blue) and control condition (red). Explanation of the variances in percent of the most influential principal components in brackets. The two most influential loadings for both principal components are included as grey arrows (Uniprot IDs). (**B**) Gene Ontology clustering of protein classes according to the PANTHER classification tool. Out of the 73 proteins which were identified as more abundant under simulated microgravity, 40 were assigned to protein classes (red). 17 hits were annotated for the 46 less abundant proteins (blue).

Out of the 3085 protein entries in the Uniprot FASTA file for *D. radiodurans*, 2168 were identified in at least one replicate (Table S1). A *Welch’s* t-test identified 119 proteins as significantly different abundant between cells grown under clinorotation (Fig. 1) and the static 1 g control cells (Fig. S1, Table S1). Out of these, 46 were less abundant and 73 were more abundant when grown in simulated microgravity. Subsequently, plotting the proteins which were identified in every replicate (1618 proteins) on a PCA (principal component analysis) was performed (Fig. 2A). Cells exposed to simulated microgravity showed a decreased spreading on PC1, which explains 32.92% of the variance in the data.

Proteins which showed an increased abundance after growth in simulated microgravity and proteins which showed a reduced abundance were uploaded to the GO (gene ontology) classification tool PANTHER (Protein Analysis Through Evolutionary Relationships) to categorize protein classes^46^. The protein class search algorithm identified 40 hits for higher abundant proteins and 17 hits for less abundant proteins. This analysis showed that hydrolases and transferases are more abundant in *D. radiodurans* grown under simulated microgravity (Fig. 2B).

**Figure 3:**
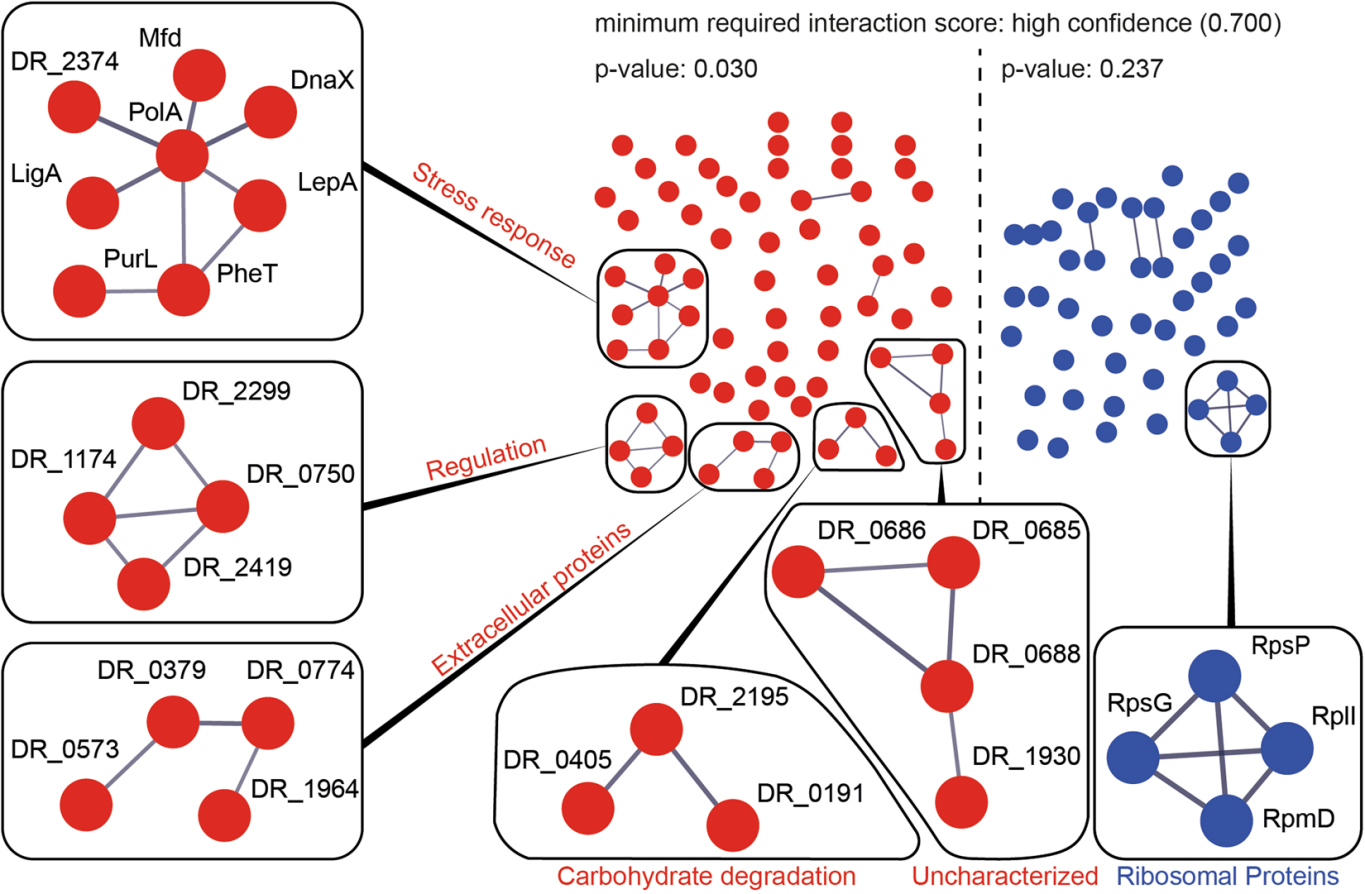
STRING database analysis of proteins higher abundant (red) and less abundant (blue) after growth in simulated microgravity. Protein-protein interactions with high confidence scores (0.700) are represented as nodes. A high p-value indicates that the number of nodes can be a result of coincidence. Protein-protein interactions with at least three proteins are emphasized.

Additionally, an analysis with the STRING (Search Tool for the Retrieval of Interacting Genes/Proteins) database^47^ revealed a significant amount of protein-protein high confidence interactions of proteins which were higher abundant after growth in simulated microgravity (Fig. 3). Lower abundant proteins on the other hand, do not show a significant amount of interactions. Protein clusters with at least three proteins were further investigated.

**Figure 4:**
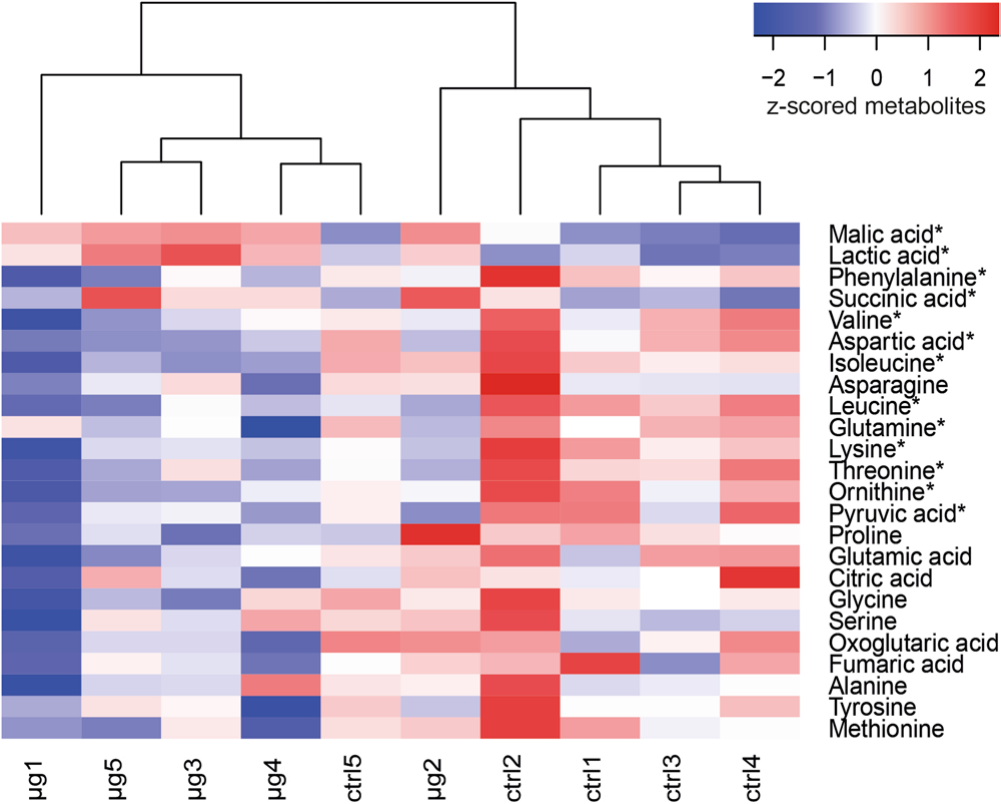
Heat map with the corresponding dendrogram of the targeted metabolomics approach. Metabolites were normalized to protein the amount of extracted proteins and z-scored. The plot was created in R with the heatmap.2 function included in the gplots package. Metabolites, which show a significantly different abundance between control and simulated microgravity samples are indicated with an asterisk (*).

Furthermore, protein clusters with high amount of protein-protein interactions identified by the STRING database were uploaded to the SMART (Simple Modular Architecture Research Tool) database. SMART is able to identify and annotate protein domains and analyse protein domain architectures^48^. Especially regulatory proteins (Fig. 4) showed a high level of conserved regions. Growth under simulated microgravity elevated the abundance of proteins with recognition domains, such as PAS (Per-Arnt-Sim) and PAC. Furthermore, these proteins contain signal transducer domains, for instance tetratrico peptide repeat (TPR) and the counterparts GGDEF and EAL which respond to certain environmental conditions to optimize gene expression. Ultimately, two of the regulatory proteins identified as higher abundant in cells, grown under simulated microgravity, harboured histidine kinase domains.

### Metabolite profiling

**Figure 5:**
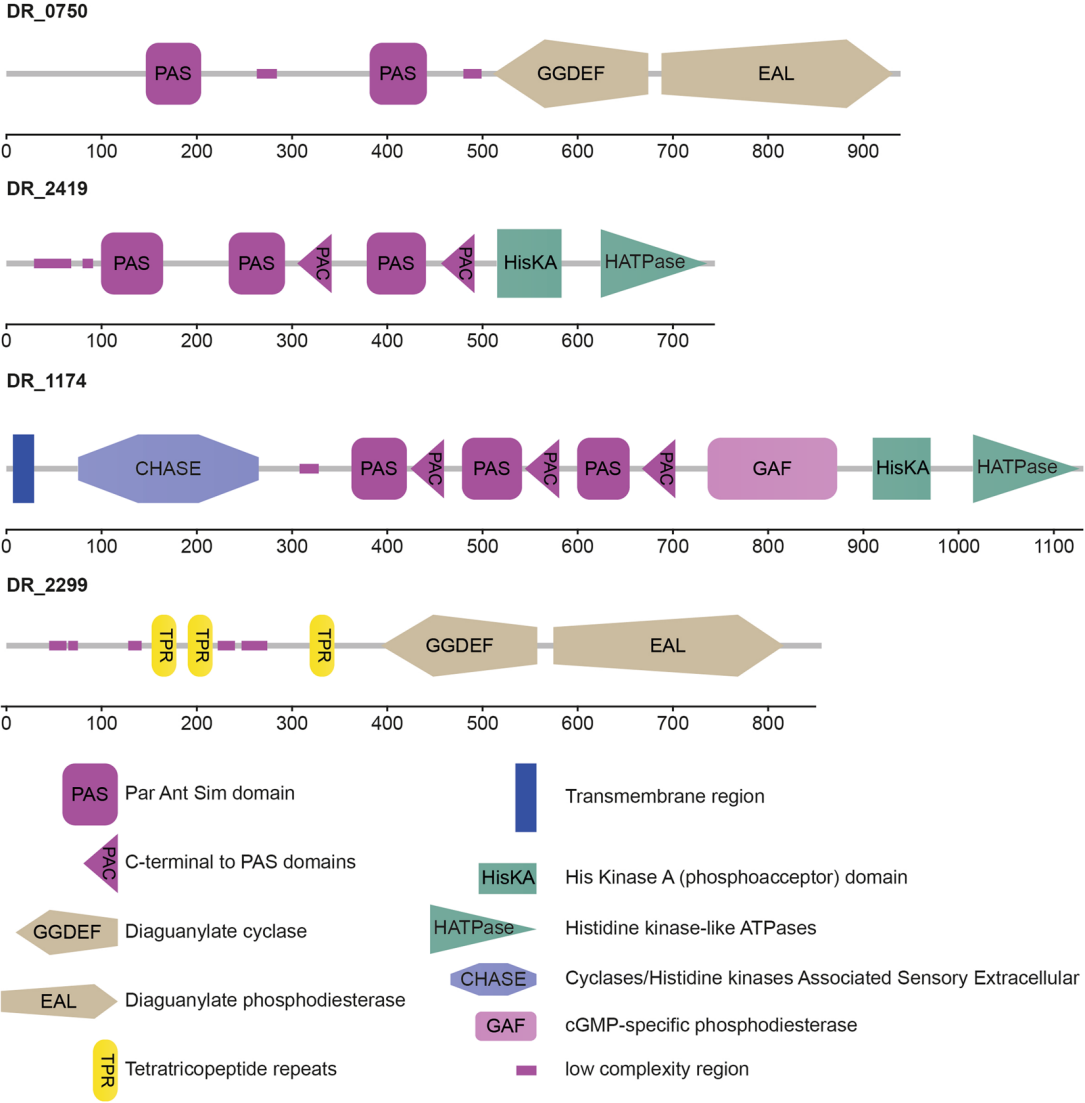
Group of signal transduction proteins that was uploaded to the SMART database to identify domains. Shows all identified domains and the corresponding length of the protein in base pairs. Identified domains in alphabetical order: CHASE (cyclase/histidine kinases associated sensory extracellular), EAL (diaguanylate phosphodiesterase), GAF (cGMP-specific phosphodiesterase), GGDEF (diaguanylate cyclase), HisKA (His Kinase A), HATPase (histidine kinase-like ATPase), PAC (C-terminal to PAS), PAS (Par Ant Sim domain), TPR (tetratricopeptide repeats)

In total, 24 metabolites were identified and quantified in the targeted approach (Table S2). Most metabolites identified by our targeted approach appeared more abundant in 1 g cells (Fig. 5). However, some TCA cycle intermediates, which are malic acid, lactic acid and succinic acid are more abundant in colonies which were grown under simulated microgravity. The alteration of TCA cycle metabolites was already observed during our previous studies, where *D. radiodurans* cells were recovered after exposure to different extreme environmental conditions^49,50^. Furthermore, the amount of amino acids appears to be reduced whenever *D. radiodurans* cells experience environmental conditions that require a certain response from the cell. Apparently, growth under simulated microgravity causes an increased demand for amino acids. Amino acids are the preferred carbon source of *D. radiodurans*^51^ and might serve as energy source during stress recovery^49^.

## Discussion

Although some studies were performed on gene expression changes after simulated microgravity in microorganisms, such as *Streptococcus* mutants^52^, *E. coli*^53^, *Bacillus cereus*^54^, *Cupriavidus metallidurans*^55^ and *Staphylococcus aureus*^56^, this is the first study that presents alterations in the proteome of *D. radiodurans* induced by simulated microgravity. The overall changes in the proteome can be observed on principle components of the PCA, as cells exposed to simulated microgravity show a reduced spreading compared to the control cells (Fig. 2A). When subjected to a specific parameter as reduced gravity, cells focus to adapt to this environmental change and therefore protein synthesis is aimed to tackle a specific task, which is visible as minimal spreading on the PCA (Fig. 2A). A similar result was shown after exposure of *D. radiodurans* cells to vacuum stress, which showed the same effects on the PCA^50^ at the level of proteins.

### General adaptation mechanisms induced by microgravity

The growth of *D. radiodurans* under simulated microgravity increased the abundance of several proteins associated with processes involving DNA, such as DR_2410 (DnaX), DR_1707 (PolA), DNA ligase DR_2069 (LigA) and the transcription repair coupling factor DR_1532 (Mfd) (Fig. 3). Among them, DnaX is the only differently abundant subunit of the DNA polymerase III complex, a multichain enzyme responsible for most replicative synthesis in bacteria^57^. Another protein that was identified as higher abundant after microgravity exposure is PolA, a polymerase with 5′-3′ exonuclease activity, which is essential for an efficient DNA repair in *D. radiodurans*, for instance after heavy ionizing radiation exposure^58^. PolA primarily fills DNA gaps that arise during replication, excision repair and recombination^59^. Previous research data suggests a connection between simulated microgravity and the induction of DNA damage and stress response in human retinal pigment epithelial cells^60^. In accordance, an induced expression of stress response genes was observed in *E. coli*, grown under modelled reduced gravity conditions^61^. A separate transcriptomic study, performed on *E. coli*, subjected to simulated microgravity in a clinostat resulted in an increased expression of genes involved in stress response and DNA replication^53^.

A proposed model, based on *E. coli* data from seven different shuttle flights describes an altered expression of genes directly and indirectly involved in glucose catabolism pathways^62^. In *Serratia marcescens*, spaceflight exposure induced alterations in genes and proteins associated with degradation and metabolism involving catabolic processes like glycolysis^63^. In our study, annotating catalytic activities of higher and lower abundant proteins after exposure to simulated microgravity revealed a distinctive peak for hydrolases (Fig. 2B). The upregulation of proteins with hydrolase activity was already observed in human mesenchymal stem cells under simulated microgravity^64^. In our work, proteins included in this group are annotated as glycogen debranching enzyme DR_0191, alpha-dextran endo-1, 6-alpha-glucosidase DR_0405, maltooligosyltrehalose synthase DR_0463 and the putative serine protease Acyl-peptide hydrolase DR_0165. Computationally derived database annotation of proteins often relies on orthologues and no experimental characterization of the proteins was performed for the used microorganism. There is a probability that the protein in the microorganism obtains the annotated or a similar function, however, it needs to be further experimentally investigated using biochemical and molecular biology tools. The higher abundances of this group of proteins with putative hydrolase activity, observed in *D. radiodurans* when grown in simulated microgravity, may help with nutrient utilization to adapt to the stress caused by the extraordinary circumstances.

### Cell envelope-associated events

**Figure 6:**
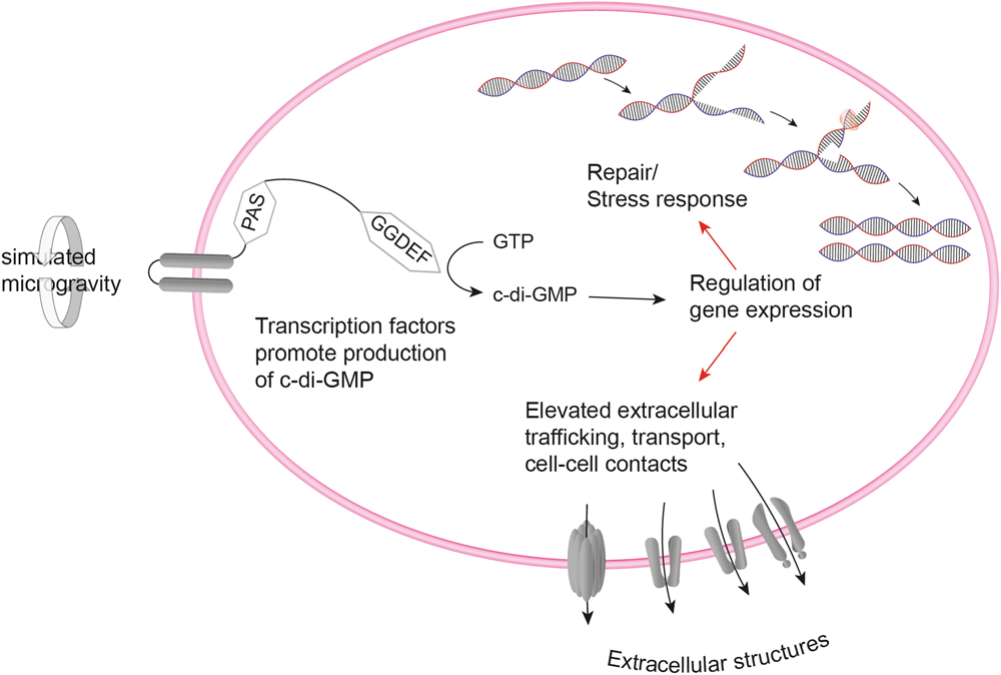
Model of the main molecular responses of *D. radiodurans* to growth under simulated microgravity. The reduced gravity is recognized by the cells through PAS/PAC regions of signal transduction proteins, which trigger GGDEF domains. Those increase the production of c-di-GMP, which affects gene expression to support DNA replication and production of extracellular structures.

Many microbial species, grown under low fluid shear environments, either in real spaceflight missions or in simulated conditions, showed an increase in extracellular polymeric substances (EPS), cell aggregation, cell-cell contacts and biofilm formation^16,65,66^. A microgravity simulation study with *R. rubrum*, cultivated in a rotating wall vessel identified several proteins belonging to cell envelope biogenesis/outer membrane as higher abundant^67^. In our study, the STRING database analysis identified a cluster of proteins associated with the extracellular milieu as higher abundant after growth in reduced gravity in *D. radiodurans* (Fig. 3). As part of the general secretion pathway, DR_1964 contribute to the secretion of unfolded proteins. In addition, the outer membrane protein DR_0379, which contains a PORTA (polypeptide transport associated) domain was identified as higher abundant after growth in simulated microgravity^68^. DR_0573, a protein specific to *Deinococcus* spp.^69^, was identified as higher abundant when grown under simulated microgravity. According to BLAST (Basic Local Alignment Search Tool), DR_0573 is an orthologue to an autotransporter outer membrane protein from *Deinococcus actinosclerus*^70^. Finally, the type IV piliation system protein DR_0774 showed an increased abundance in *D. radiodurans* after growth in simulated microgravity. This protein was identified as a secretin like S-layer component of *D. radiodurans*^71^. DR_0774 and DR_2577 (SlpA), the most representative protein of the *D. radiodurans* cell wall, could contribute to a complex that could span both the inner and the outer membranes. DR_0774 is acting as a structural pillar that brings stability to the plane of the outer membrane^71^ which is also the main channel through which trafficking is managed^72^. A high abundance of these channels may contribute to increased extracellular trafficking, cell-cell contacts and other cell envelop-associated events in *D. radiodurans* as a consequence of growth under simulated microgravity (Fig. 6). Lastly, protein DR_2299, which was more abundant under simulated microgravity growth, contains TPR regions which basic function is to mediate protein-protein interactions and the assembly of multiprotein complexes^73^. Such complexes have been shown to fulfil important roles in biofilm formation in *Bacillus subtilis*^74^ and may influence EPS production and/or membrane-associated events in organisms grown under reduced gravity.

### Transcriptional regulation

Previous studies emphasized the importance of the Hfq regulon in various bacterial species as response to simulated or real microgravity conditions^20,36,75^. This regulon is missing in *D. radiodurans*^76^, however comparing the obtained data (Table S1) to a previous spaceflight proteomics study performed on *P. aeruginosa* revealed in both cases a decreased abundance of ribosomal proteins^21^. Another microgravity simulation study, using a high-aspect rotating vessel, and a subsequent transcriptomic approach confirmed the lower abundance of ribosomal proteins in *Streptococcus mutans*, grown for 8 h under reduced gravity^52^. The same study showed an increase in expression of genes encoding transcriptional regulators for cells, grown under simulated microgravity. Furthermore, RNA seq of spaceflight exposed *S. aureus* and (to a lesser extent) *B. subtilis* showed a decreased expression of proteins involved in folding and associated processes^77^. Similar results were obtained by subjecting *Mycobacterium marinum* to simulated microgravity in a high aspect ratio vessel. A subsequent transcriptomic approach induced a reduction in the expression of proteins involved in translation^55^. In yeast, the transcriptional regulator Rap1p is responsible for the expression of many genes, including ribosomal proteins and those whose expression is altered in response to changes in growth rate^78^. Similar to Hfq or Rap1p, other regulatory proteins must be responsible for the response of *D. radiodurans* to reduced gravity conditions. A cluster of proteins which are related to signal transduction was identified throughout our analysis. In order to evaluate these proteins in more detail, the sequences were uploaded to the SMART database, which is able to identify and annotate genetically mobile domains (Fig. 4). Two of the identified proteins (DR_0750 and DR_2299) contain GGDEF domains, conserved regions which are detected in many prokaryotic proteins, often in various combinations with sensory regulatory components^79,80^. Its function is to act as diguanylate cyclase to catalyse cyclic (c)-di-GMP production, which is used as intracellular signalling molecule to control multicellular behaviour^81^. This includes biosynthesis of exopolysaccharides, formation of biofilms and regulation of gene expression^82^. In *P. aeruginosa* WspR, a GGDEF-type response regulator is correlated to auto aggregation^83^. Enhanced cell aggregation and clumping was observed for *S. enterica* Typhimurium cultured in space^75^ as well as for *Candida albicans* during a short-term spaceflight^21^. After growth in simulated microgravity, assuming to be achieved by high-aspect-ratio vessels, several bacterial cultures tend to build self-aggregative biofilms^52,56,84^. It is assumed that differences in the EPS are responsible for cellular aggregation^85^. Although *D. radiodurans* R1 does not produce biofilms, it is likely that the extracellular trafficking and cell envelope-associated processing are altered during growth under reduced gravity (Fig. 6). Recent investigations report on biofilm formation of a minor genetically modified *D. radiodurans* strain^86^, although these investigation requires further critical assessment. To regulate the level of c-di-GMP, the EAL domain (can be activated if necessary) in the same protein is used, a diguanylate phosphodiesterase to break the phosphodiester bond, as high levels of c-di-GMP are toxic to the cells^87^. Together these messenger domains are assumed to be involved in modulating cell surface structures^88^ and extracellular protein production^79^.

Another domain, which appear in three of the identified proteins (DR_0750, DR_2419 and DR_1174) is the PAS domain, a common signalling sensor in signalling proteins in all kingdoms of life^89^. Transduction of redox signals might be a common way to sensor by PAS domains, which are always located intracellularly. However, they are able to monitor the external milieu by detecting changes in the electron transport system^90^. PAS domains are often supported by PAC motifs, which occur C-terminal to many PAS domains and may contribute to PAS folding^91^. Certain environmental conditions, such as the availability of nutrients and oxygen can trigger biofilm dispersal^92^. In *P. aeruginosa*, this is enabled through a protein (RbdA), containing PAS and PAC regions as sensory domains as well as GGDEF and EAL domains. Under stressful conditions, the phosphodiesterase (EAL) domain of RbdA is active and catalyses the cleavage of the second messenger c-di-GMP, which ultimately leads to biofilm dispersal^93^. Although the enzymology behind c-di-GMP synthesis and degradation has been elucidated, the detailed mechanism through which it operates and how EPS and secretion processes are affected, remain obscure^94^.

Additionally, cells grown under simulated microgravity showed elevated amounts of transferases (kinases, methyltransferases, glycosyltransferases and phosphorylases). Those (Fig. 2B) might be responsible for transcriptional regulations and post-translational modifications as a response to the altered environmental condition. Apart from that, two high abundant proteins (DR_1174 and DR_2419) contain histidine kinase domains, key elements in two-component signal transduction systems which control complex cellular processes^95^ and are involved in adapting to environmental changes^96^.

## Conclusion

This study under simulated microgravity conditions using 2D clinorotation was performed with respect to experimental exposure of *D. radiodurans* outside the ISS. Apart from radiation and vacuum, it is important to understand the molecular response to microgravity as one exceptional environmental factor present in outer space. However, considering that our study is performed under simulated conditions, further verification might be necessary in real microgravity in space. Most studies regarding microbial response to real microgravity focus on pathogens and biofilm forming bacteria. The growth of *D. radiodurans* under simulated microgravity obviously induce signal proteins responsible for the additional production of proteins connected to the extracellular milieu and cell envelope-associated events. The reduced gravity environment is recognized by PAS (and PAC) regions, which activate GGDEF to catalyse the production of c-di-GMP. As a result, *D. radiodurans* produces more proteins associated with the extracellular region, whereas in other EPS-forming microorganisms, biofilm production is increased. Other protein domains, e.g., TPR and GGDEF, convey the obtained signal to influence gene promotors. Therefore, the abundances of proteins which are responsible for DNA processing and extracellular membrane-associated events are increased and ultimately, those lead to a prolonged exponential phase and elevated extracellular trafficking (Fig. 6). Although results obtained from the applied approaches indicate essential components for the response of *D. radiodurans* to simulated microgravity, future studies are needed to validate the hypotheses. Computationally derived results in our study need to be further critically assessed by thorough biochemical analysis of targeted proteins. To verify enzymatic switches that respond to gravity stress, a comparison to cells grown under increased gravity is advised. Furthermore, the generation of mutant strains for identified signal transducer proteins combined with other methods such as a transcriptomic approach and various electron microscopy-based techniques can help to completely unravel the molecular mechanisms in *D. radiodurans* responsible to adapt to simulated microgravity conditions.

## Methods

### Strain, media and storage

A bacterial stock of *D. radiodurans* R1 (ATCC 13939, 1 × 10^8^ CFU/ml) was stored at −80 °C in 1:1 glycerol and 2x TBG-broth (1% tryptone (w/v), 0.6% beef extract (w/v), 0.2% glucose (w/v). For recovery and pre-cultures, 1x TBG was inoculated with 10 µl of a 10^8^ CFU/ml frozen stock solution and incubated at 37 °C for 15 h. For inoculating agar plates, 10 µl of a PBS-washed (0.7%, Na_2_HPO_4_ · 2 H_2_O (w/v), 0.4% NaCl (w/v), 0.3% KH_2_PO_4_ (w/v), pH 7.4) pre-culture were pipetted on 1x TBG (solidified with 1.5% (w/v) agar) and incubated at 37 °C for 2 days (Fig. 1).

### 2-D clinostat: simulation of microgravity

For the simulation of microgravity, a commercially available fast-rotating 2-D clinostat (UN-KTM2, Advanced Engineering Services, Japan) was used. The sample holder was slightly modified, to hold up to eleven petri-dishes. TBG-medium was poured in germ-counting petri-dishes (Greiner Bio-One GmbH, Germany), which were labelled with a grid on the backside of the plate. The centre of the grid represented the centre of the petri dish and therefore marked the inoculation position of the respective plate. Each plate was inoculated with 10 µl of a fresh PBS-washed overnight culture and air-dried for 2 min. Plates were wrapped with Parafilm to prevent contaminations and mounted in the 2-D clinostat, secured by a lid and four butterfly screws. The clinostat was placed in a standard laboratory incubator at 37 °C. The rotation axis was aligned parallel to the ground with a spirit level. The clinostat was set to 60 rpm, which correspond to ~0.0161 g residual acceleration assuming a final colony diameter of ≤0.8 cm (~r ≤ 0.4 cm). Control cells were placed at a similar position near the clinostat to mimic similar temperature and humidity conditions, however kept at static 1 g control.

### Colony harvesting

After incubation for 48 h, colonies were immediately scratched off the agar by using a sterile 5 µl plastic loop and directly transferred into ice-cold PBS-buffer. For every biological replicate, five individual colonies were pooled to one combined sample (n = 5). To secure reproducibility of the results, three clinostats were used at different time points. Combined samples were washed two times in ice-cold PBS-buffer at 4 °C. The supernatant was discarded, and the pellet was frozen in liquid nitrogen. Dry ice was used to transport the samples until the samples could be stored at −20 °C.

### Extraction of proteins and metabolites

The integrative extraction of proteins and metabolites was performed as described before (27, 31). Approximately 0.5 g lysing matrix B (MP Biomedicals) and 750 µL ice-cold MCW (methanol: chloroform: water 2.5:1:0.5) were added to the frozen cell pellets. Homogenization was performed in a FastPrep 24 instrument (MP Biomedicals; 5 * 30 s, 6.5 ms^−1^; cooled on ice between circles), followed by 15 min incubation on ice. Samples were centrifuged (21000 g/4 min/4 °C) and the supernatants were transferred in new tubes for subsequent metabolite purification. A second extraction of the pellets was performed with 250 µL MCW. Samples were vortexed, 5 min incubated at room temperature (RT), centrifuged (21000 g/4 min/4 °C) and the supernatant was transferred to the tubes for metabolite purification. H_2_O (300 µL) was added to the supernatants to achieve a phase separation. After centrifugation (21000 g/4 min/4 °C), the upper polar phases were transferred to new tubes, carefully dried in a vacuum concentrator (ScanVac, Labogene) and stored at −20 °C until derivatization.

### Protein purification and digestion

Protein pellets were washed with 1 mL methanol (MeOH), centrifuged (21000 g/5 min/4 °C) and air-dried within a laminar flow hood for 10 min. Pellets, containing nucleic acids and proteins were solubilized in 1 mL TRIzol. Together with the lysing matrix B, samples were homogenized using the bead beater one more time (30 s/6.5 ms^−1^). Afterwards, samples were incubated 5 min on a turning wheel (20 rpm) at RT. To separate phases, 200 µL chloroform were added, samples were incubated for 3 min on a turning wheel (10 rpm) and centrifuged (21000 g/4 °C/15 min). The lower, apolar, protein containing phases were transferred to new tubes. They were washed with 550 µL H_2_O, incubated 3 min on a turning wheel (20 rpm), centrifuged (21000 g/4 °C/5 min) and the lower, apolar phase was transferred to new tubes. For overnight precipitation at −20 °C, 1.5 mL 0.1 M NH_4_Ac in MeOH (containing 0.5% β-mercaptoethanol) were added to each sample. Proteins were subsequently centrifuged (10000 g/15 min/4 °C) and the supernatants were discarded. The pellets were washed with 1 mL acetone, followed by disruption of the pellets in a ultrasonication bath for 5 min. Samples were centrifuged (10000 g/5 min/4 °C) and the supernatants were discarded. The washing procedure was repeated one time with 1 mL acetone and one time with 90% acetone. After the final washing step, samples were air dried for 15 min under a laminar flow hood.

Protein pellets were solubilized in 40 µL 8 M urea/4% SDS (sodium dodecyl sulphate) and the total protein concentration was estimated with a bicinchoninic acid assay (BCA) against bovine serum albumin (BSA) (Fig. S2). 80 µg proteins, mixed with Laemmli buffer (Bio-Rad) for each replicate were loaded on SDS-polyacrylamide gels (separation gel 12%, stacking gel 5%). Samples were run through the stacking gel with a voltage of 40 V, which was increased to 80 V once the samples reached the separating gel. After the bromophenol blue run approximately 1 cm into the separating gel, the electrophoresis was stopped. The gel was stained with 40% MeOH, 10% acetic acid, 0.1% Coomassie R-250 in milliQ-H_2_O for 30 min. Destaining was performed 4 times (20 min) with 40% MeOH, 2% acetic acid, followed by washing the gel in H_2_O for 30 min.

The protein bands for each sample were cut out of the gel and further cut into small pieces (of approximately 1 mm^3^). To destain protein bands, 1 mL 25 mM ammonium bicarbonate (AmBic) in 50% acetonitrile (ACN) was added to each sample. Samples were incubated on a thermal shaker (650 rpm/15 min/37 °C) and the supernatants were discarded. This procedure was repeated two times until the blue colour disappeared from the pieces. To dry samples, 300 µL ACN were added, incubated 5 min at RT and the supernatants were discarded. Next, disulphide bonds were reduced with 20 mM dithiothreitol (DTT) in 100 mM AmBic (650 rpm/30 min/37 °C) and the supernatants were discarded. Gel pieces were washed with ACN and alkylation of reduced cysteine residues was performed with 55 mM iodoacetamide (IAA) in 100 mM AmBic (60 min/RT). Gel pieces from each sample were washed with 25 mM AmBic in H_2_O, 25 mM AmBic in 50% ACN and in 100% ACN (650 rpm/15 min/37 °C). Proteins were digested by covering them with trypsin (12.5 ngµL^−1^, in 25 mM AmBic, 10% ACN, 5 mM CaCl_2_) for 16 h.

To extract peptides from each sample, 150 µL of 50% ACN including 1% formic acid (FA) were added. Samples were incubated for 5 min at RT, briefly sonicated and transferred into a new tube. The procedure was repeated once with 50% ACN (1% FA) and one time with 90% ACN (1% FA). Collected supernatants were dried down in a vacuum concentrator.

To desalt samples, peptides were suspended in 4% ACN (0.25% FA), incubated at RT and centrifuged (21000 g/2 min/4 °C). The desalting C18 spec plate (Agilent) membranes, connected to a water jet pump, were activated with 2 × 800 µL MeOH and washed with 2 × 800 µL H_2_O. Samples were loaded on the membranes and incubated for 10 min at RT (only gravity). Peptides were first washed with 2 × 800 µL H_2_O and finally eluted with 3 × 800 µL MeOH. Collected samples were dried in a vacuum concentrator.

Samples were resuspended in 100 µL 2% ACN (0.1% FA) and the total peptide concentration was estimated with a colorimetric peptide quantification assay (Pierce) (Fig. S2). The peptide concentrations were adjusted to 50 ng/µl for LC-MS/MS analysis.

### HPLC nESI MS/MS

For shotgun proteomics measurements, 5 µL of each sample were injected into an nHPLC-Orbitrap QExactive (Thermo Fisher Scientific, Bremen, Germany), measurement settings were described before^49^. Data analysis was performed with Maxquant^97^. The minimum peptide length for identification was set to 7 amino acids and one unique peptide was required for protein identification (FDR 1%, based on target decoy database). For identification, measured spectra were compared to the *D. radiodurans* FASTA file from Uniprot (January 2018, 3085 sequences in the database). Further Maxquant settings: 20 ppm first search peptide tolerance, 4.5 ppm main search peptide tolerance, maximum of 2 missed cleavages, maximum number of 5 modifications per peptide (variable: oxidation (M) and acetylation of protein N-term, fixed: carbamidomethylation (C)), label free quantification of samples.

### Derivatization and analysis of the metabolites with GC-TOF-MS

Polar metabolites were dissolved in 10 µL of 40 mg mL^−1^ methoxyamine-hydrochloride in pyridine through shaking at 650 rpm at 30 °C for 90 min. Subsequently, 40 µL of a silylation mix (1 mL N-methyl-N-trimethylsilyltrifluoroacetamid spiked with 30 µL of a mix of even-number alkanes (C10-C40)) was added and the mixture was incubated for 30 min at an agitation rate of 650 rpm at 37 °C. After centrifugation (14000 g, 2 min), the supernatant was transferred into a glass vial and 1 µL of it was injected into the GC (Agilent 6890 gas chromatograph) in splitless injection mode.

For separation of the metabolites, an Agilent HP-5MS column (30 m length, 0.25 mm diameter and 0.25 μm film) was used. Further parameters were set as following: flow rate 1 ml/min; injection temperature 230 °C; column temperature started at 70 °C for one minute, then heated up to 330 °C in 9 min, where it was hold for 8 min; recorded masses in the LECO Pegasus 4D GC × GC-TOF spectrometer were set between 40–700 m/z. Apart from the samples, a house intern standard mix of certain metabolites was measured to get level 1 identifications of common primary metabolites.

Identifications of the metabolites were based on matching the obtained MS-spectra and retention times with an in-house library (extended gmd database). Peak integration was performed with the LECO ChromaTOF software.

### Statistical analysis

Data processing of proteomic and metabolomic measurements was performed similarly. The peptide content was normalized before measurement; therefore 250 ng peptides were injected for each replicate. Consequently, the LFQ (label free quantification) intensity results for each identified protein sequence were used for relative quantification without any further normalization steps. However, peaks derived from the metabolite measurements were normalized to the protein content measured by the BCA (Fig. S2) of each individual replicate. A *Welch’s* t-test was performed to identify proteins and metabolites of interest (p-value below 0.05). For the STRING analysis, proteins of interest that were higher and lower abundant after simulated microgravity exposure were uploaded independently. The protein-protein interaction analysis was performed on high confidence level (0.700), to minimize false positives.

## Supplementary information

Supplementary Figures and Tables Captions

Table S1

Table S2

## Data Availability

All data generated or analysed during this study are included in this published article (and its supplementary information files).
