# Supplementary material for: Molecular response of Deinococcus radiodurans to simulated microgravity explored by proteometabolomic approach
Source: Sci Rep. 2019 Dec 5;9:18462. doi: 10.1038/s41598-019-54742-6 (PMC6895123; doi:10.1038/s41598-019-54742-6)
Supplement: Supplementary file 1 — Supplementary Figures and Tables Captions [file 41598_2019_54742_MOESM1_ESM.pdf]

Molecular response of *Deinococcus radiodurans* to simulated microgravity explored by proteometabolomic approach

Emanuel Ott, Felix M. Fuchs, Ralf Moeller, Ruth Hemmersbach, Yuko Kawaguchi, Akihiko Yamagishi, Wolfram Weckwerth and Tetyana Milojevic

*Corresponding author
tetyana.milojevic@univie.ac.at

Supplementary Information:

Supplementary Figure 1:	Heatmap of normalized LFQ intensities from significantly different expressed proteins identified in every replicate.
Supplementary Figure 2:	Results of protein and peptide quantification photometric assays for sample normalization.
Supplementary Table 1:	Raw protein LFQ intensities, corresponding statistical analysis, number of identified peptides and calculated Maxquant score.
Supplementary Table 2:	Targeted metabolomics approach with normalized peak areas and corresponding statistical analysis.

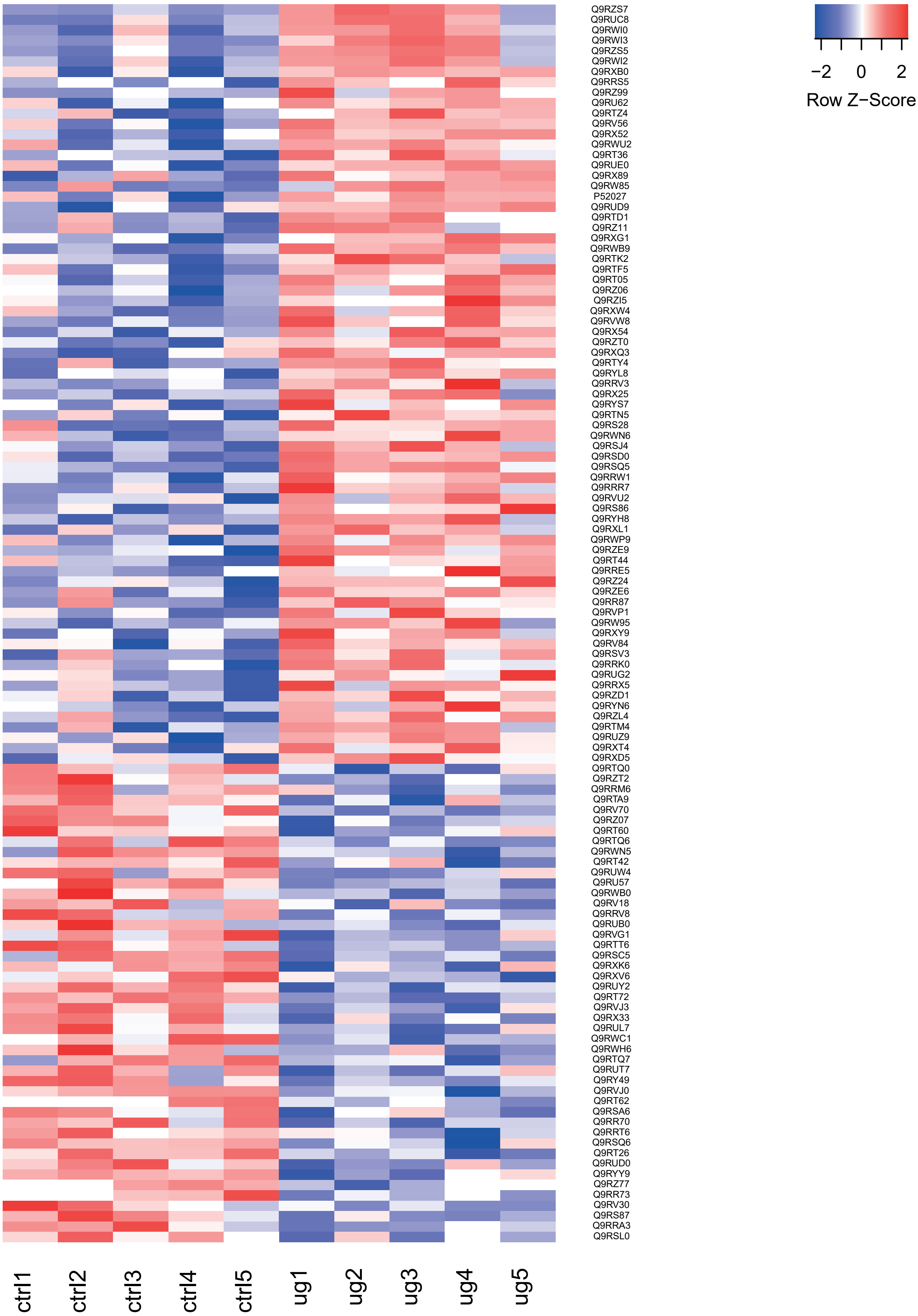

Figure S1: Heatmap of normalized (z-scored) LFQ values of significantly different expressed proteins identified in every replicate. The minimum requirement for an identification was one unique peptide.

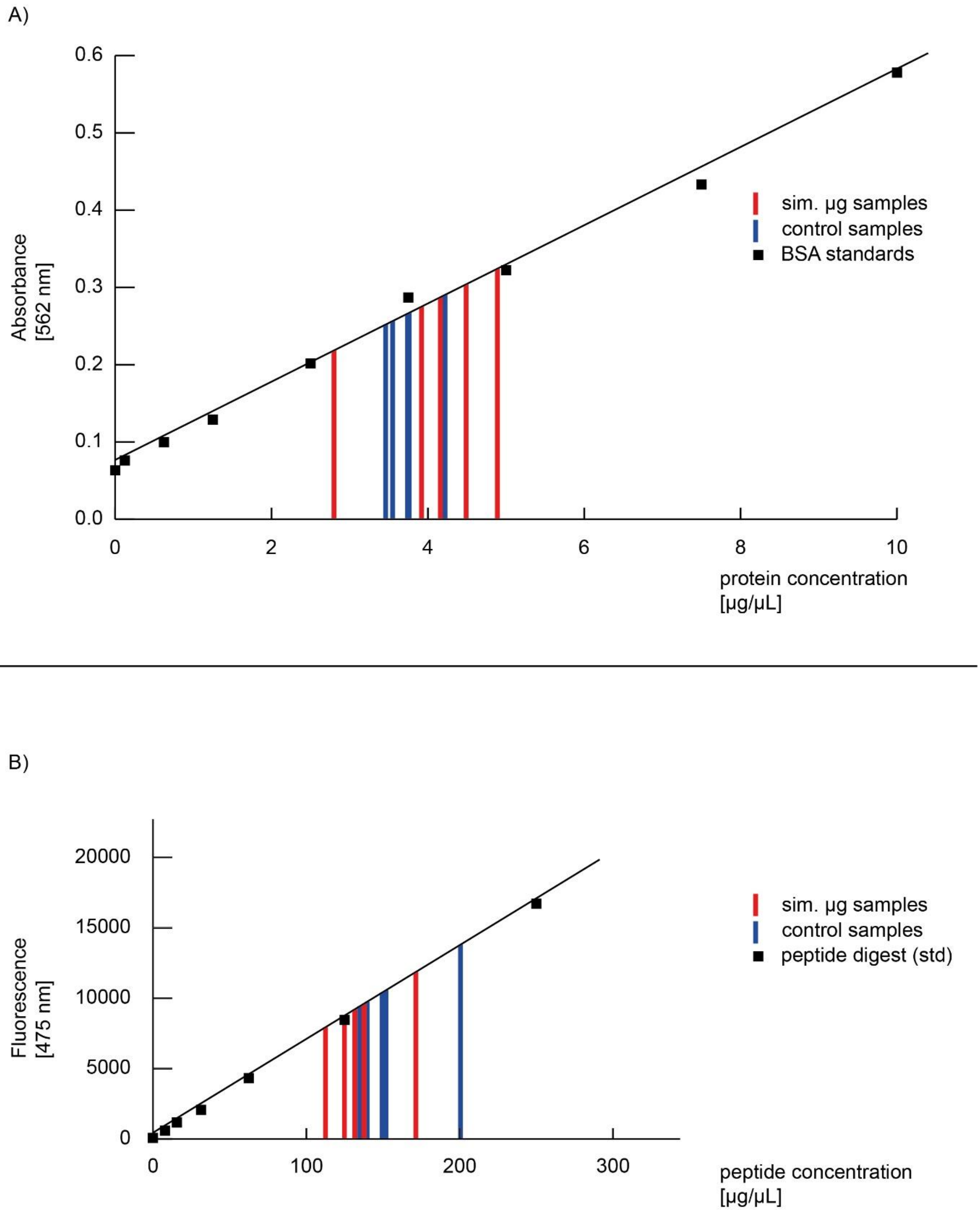

Figure S2 A) Protein concentration in µg/µL of each replicate (1:5 diluted) against a BSA standard in different concentrations. B) Peptide concentration in ng/µL of each replicate, measured against a standard peptide digest solution.
